# Serosurvey in BNT162b2 vaccine-elicited neutralizing antibodies against authentic B.1, B.1.1.7, B.1.351, B.1.525 and P.1 SARS-CoV-2 variants

**DOI:** 10.1080/22221751.2021.1940305

**Published:** 2021-06-18

**Authors:** Alberto Zani, Francesca Caccuri, Serena Messali, Carlo Bonfanti, Arnaldo Caruso

**Affiliations:** Section of Microbiology, Department of Molecular and Translational Medicine, University of Brescia, Brescia, Italy

**Keywords:** SARS-CoV-2, variant of concern, variant of interest, BNT162b2 vaccine, neutralizing antibodies

## Abstract

In this study, we show that BNT162b2 vaccine-elicited antibodies efficiently neutralize SARS-CoV-2 authentic viruses belonging to B.1, B.1.1.7, B.1.351, B.1.525 and P.1 lineages. Interestingly, the neutralization of B.1.1.7 and B.1.525 lineages was significantly higher, whereas the neutralization of B.1.351 and P.1 lineages was robust but significantly lower as compared to B.1 lineage. Following our findings, we consider that the BNT162b2 vaccine offers protection against the current prevailing variants of SARS-CoV-2.

Since the first case of coronavirus disease 2019 (COVID-19), pandemic illness has spread to millions of people globally. In response to the urgent need for COVID-19 vaccines, different candidates have been designed. Italy launched its immunization campaign at the end of December 2020 with the inoculation of the first Severe Acute Respiratory Syndrome Coronavirus-2 (SARS-CoV-2) spike-based vaccine approved in the Country, the BNT162b2 from Pfizer [1]. Since the beginning of vaccine development, different SARS-CoV-2 variants have arisen in the United Kingdom (UK, B.1.1.7 lineage) [2], South Africa (SA, B.1.351 lineage) [2], Brasil (BR, P.1 lineage) [2], and Nigeria (NI, B.1.525 lineage; https://cov-lineages.org/global_report_B.1.525.html). All these variants are characterized by multiple mutations in their spike glycoproteins raising concern over vaccine efficacy. An important issue for spike-based vaccines is whether the authentic virus can escape vaccine-elicited neutralizing antibodies. To address this question, we collected and tested a panel of human sera randomly selected from 37 volunteers obtained between 10 and 20 days after the administration of the second dose of BNT162b2, which occurred three weeks after the first immunization (Table S1). All the volunteers had no history of natural SARS-CoV-2 infection, as attested by negativity for detection of antibodies against the SARS-CoV-2 Nucleocapsid protein (Elecsys^®^ Anti-SARS-CoV-2, Roche, Indianapolis, IN, USA). Electro-chemiluminescence immunoassay (ECLIA) showed that all sera collected contained antibodies against the spike glycoprotein receptor-binding domain (RBD) (Elecsys^®^ Anti-SARS-CoV-2 S, Roche). As shown in Table S1, a broad range of reactivity profiles to RBD, ranging from 265.7 to >5000 UI/ml, were noticed. Each serum was then tested for neutralization of the wild type (B.1 lineage), UK (B.1.1.7 lineage), SA (B.1.351 lineage), BR (P.1 lineage) and NI (B.1.525 lineage) authentic viruses isolated in Italy. Neutralization was performed by cytopathic effect (CPE)-based assay [3], using viruses soon after having confirmed their identity by next-generation whole-genome sequencing (CleanPlex^®^ SARS-CoV-2 Flex Paragon Genomics, Hayward, CA, USA). Figure S1 shows key mutations in the RBD of the viral variants under study.

All the serum samples efficiently neutralized SARS-CoV-2 B.1 lineage and all the viral variants (Figure 1). In particular, as compared with neutralization of SARS-CoV-2 B.1 lineage, neutralization of SARS-CoV-2 B.1.1.7 lineage and SARS-CoV-2 B.1.525 lineage was significantly higher, and neutralization of SARS-CoV-2 B.1.351 lineage and SARS-CoV-2 P.1 lineage was robust but significantly lower. Almost all the sera neutralized each virus at titres higher than 1:40, with only 2 sera reaching neutralization activity of the SARS-CoV-2 B.1.351 lineage at a 1:10 and 1:20 dilution. A tendency to a better neutralization was observed in sera from younger people, as compared to the older ones, even if a statistically significant difference was reached only against the two more sensitive viruses to neutralization (B.1.1.7 and B.1.525 lineages) (Figure S2A). On the other end, a statistically significant difference in neutralizing all lineages was observed with sera collected between 10 and 14 (*n* = 12) than with those collected between 15 and 20 (*n* = 25) days after the administration of the second dose of BNT162b2 (Figure S2B).

**Figure 1.**
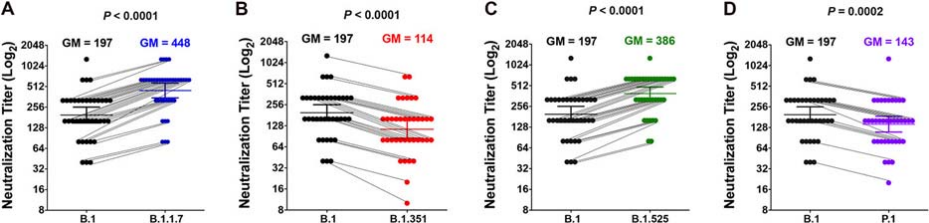
Serum neutralization of authentic SARS-CoV-2 B.1 lineage and its viral variants. (**A**) SARS-CoV-2 B.1 lineage and SARS-CoV-2 B.1.1.7 lineage. (**B**) SARS-CoV-2 B.1 lineage and SARS-CoV-2 B.1.351 lineage. (**C**) SARS-CoV-2 B.1 lineage and SARS-CoV-2 B.1.525 lineage. (**D**) SARS-CoV-2 B.1 lineage and SARS-CoV-2 P.1 lineage. Shown are the results of neutralization test with the use of 37 samples obtained from 37 volunteers between 10 and 20 days after the administration of the second dose of the BNT162b2 vaccine (which occurred three weeks after the first immunization). Neutralization of authentic viruses was performed by cytopathic effect (CPE)-based assay using a viral titre of 10^2^ TCID_50_. The neutralization titre of the serum sample was calculated as the reciprocal of the highest dilution that protected more than 50% of cells from CPE. Sera with different neutralization titre against SARS-CoV-2 B.1 lineage and viral variants are connected by lines. Horizontal lines and the numbers over the bars indicate geometric mean titres (GM). The I bars indicate 95% confidence intervals. Statistical analysis was performed using the paired *t*-test and two-tailed *P* values were calculated. The statistical significance of the difference between neutralization titres in the SARS-CoV-2 B.1 lineage and in each variant virus neutralization assay with the same serum samples are as follows: *P* < .0001 for SARS-CoV-2 B.1.1.7 lineage; *P* < .0001 for SARS-CoV-2 B.1.351 lineage; and *P* < .0001 for SARS-CoV-2 B.1.525 lineage; and *P* = .0002 for SARS-CoV-2 P.1 lineage.

Interestingly, SARS-CoV-2 B.1.1.7 lineage and SARS-CoV-2 B.1.525 lineage bearing a single mutation in the RBD (N501Y or E484K) were robustly neutralized by vaccine-elicited antibodies. These mutations leading to increased RBD affinity to ACE2 are known to favour viral transmissibility [4,5], but they likely better expose this functional epitope to neutralizing antibodies. At the same time, two close mutations in the RBD of SARS-CoV-2 B.1.351 lineage and SARS-CoV-2 P.1 lineage (E484K + N501Y) challenged antibody neutralization, possibly due to perturbation of antigen recognition. This hypothesis is supported by recent molecular dynamic data showing that the E484K or N501Y mutations alone increase the affinity of the RBD domain for ACE2, while the combination of E484K + N501Y results in the highest degree of RBD conformational alterations [5]. Limitation of the current study is the lack of correlation between neutralizing antibody titres and protection against COVID-19 disease. Considering the capability of the BNT162b2 to elicit potent T-cell immunity against multiple variants [6], vaccine-mediated protection must be validated by data on clinical effectiveness collected in regions where SARS-CoV-2 variants are common.

## Supplementary Material

Supplemental_Figure_Legends.docx
