# Supplementary material for: Serosurvey in BNT162b2 vaccine-elicited neutralizing antibodies against authentic B.1, B.1.1.7, B.1.351, B.1.525 and P.1 SARS-CoV-2 variants
Source: Emerg Microbes Infect. 2021 Jun 18;10(1):1241–3. doi: 10.1080/22221751.2021.1940305 (PMC8216260; doi:10.1080/22221751.2021.1940305)
Supplement: Supplemental_Figure_Legends.docx [file TEMI_A_1940305_SM8579.docx]

**Figure Legends**

**Figure S1.** Schematic overview of the S proteins from the SARS-CoV-2 wild-type (SARS-CoV-2 B.1 lineage) and variants under study. Locations of deletions (black lines) synonymous (blue lines) and nonsynonymous (red lines) mutations are shown. NTD denotes the N-terminal domain (14–305 residues), RBD receptor-binding domain (319–541 residues), FP fusion peptide (788–806 residues), HR1 heptad repeat 1 (912–984 residues), HR2 heptad repeat 2 (1163–1213 residues), TM transmembrane domain (1213–1237 residues) and CT cytoplasmic domain (1237–1273 residues). Genomic data for SARS-CoV-2 B.1 lineage (accession number: EPI_ISL_1379197), SARS-CoV-2 B.1.1.7 lineage (accession number: EPI_ISL_1379437), SARS-CoV-2 B.1.351 lineage (accession number: EPI_ISL_1379439), SARS-CoV-2 B.1.525 lineage (accession number: EPI_ISL_1379442) and SARS-CoV-2 P.1 lineage (accession number: EPI_ISL_873209) are available at Global initiative on sharing all influenza data (GISAID). In the lower panel, alignment and comparison among amino acid sequences of the RBD are reported. Sequences are represented by the single-letter amino acid code. Amino acid positions are referred to the SARS-CoV-2 B.1 lineage, adopted as reference for this analysis. Each amino acid residue of variants not differing from the SARS-CoV-2 B.1 lineage sequence is represented by a dot.

**Figure S2.** Analyses of neutralization titers of sera against authentic SARS-CoV-2 B.1 lineage and its variants. 37 human sera obtained by BNT162b2 vaccinated volunteers were tested against SARS-CoV-2 B.1, B.1.1.7, B.1.351, B.1.525 and P.1 lineages. **A,** shown are the results of neutralization test divided in 2 groups (n = 20 sera from younger adults, aged 23 to 49 and n = 17 sera from older adults, aged 51 to 69). **B,** shown are the results of neutralization titers divided in 2 groups according to the collection period after the administration of the second dose of the BNT162b2 vaccine (n = 12 collected between 10-14 days post vaccination; n = 25 sera collected between 15-20 days post vaccination). Horizontal lines indicate geometric mean titers (GM). The I bars indicate 95% confidence intervals. Statistical analysis was performed using the two-tailed unpaired t-test, *P* values indicate the statistical significance of neutralization titers between the two indicated groups for each SARS-CoV-2 lineage.
